# Diffuse Large B-Cell lymphoma Misdiagnosed as a Hematoma: Case Report

**DOI:** 10.3390/medicina59101775

**Published:** 2023-10-05

**Authors:** Jae Hyun Lee, Jiyoung Yun

**Affiliations:** Department of Plastic and Reconstructive Surgery, Busan Paik Hospital, School of Medicine, Inje University, Busan 47392, Republic of Korea; drchaser2@gmail.com

**Keywords:** lymphoma, large B-cell, diffuse, hematoma

## Abstract

*Background:* Diffuse large B-cell lymphoma (DLBCL), the most common subtype of non-Hodgkin’s lymphoma, often presents diagnostic challenges due to its diverse clinical presentation. We present a case of DLBCL that was initially misdiagnosed as a hematoma, highlighting the importance of considering malignancy when faced with unresponsive soft tissue swelling. *Methods:* A 76-year-old man presented to the emergency department with right periorbital swelling and ecchymosis following a traumatic injury. Despite ongoing anticoagulant therapy (warfarin) for atrial fibrillation, the symptoms persisted. A CT scan of the facial bones revealed a large, irregular, homogeneous mass. Initially, the clinical history and radiologic findings suggested an extraconal hematoma. As a result, an incision and drainage procedure was performed, and the old blood was evacuated. However, the patient’s symptoms continued to worsen. A follow-up CT scan showed enlargement of the lesion, prompting a surgical excisional biopsy. *Results:* Pathologic examination of the excised mass revealed a diffuse infiltrate of lymphocytes surrounding the tissue, confirming the diagnosis of diffuse large B-cell lymphoma (DLBCL). The patient was subsequently referred to hematology for further management. *Conclusions:* Although rare, DLBCL is associated with a challenging prognosis. This case highlights the diagnostic complexities that can arise, particularly when factors such as prior injury and anticoagulant therapy confound the clinical picture. The initial misclassification of the condition as a hematoma led to a delay in diagnosis and the subsequent initiation of treatment. Therefore, it is imperative to remain vigilant and consider malignancy as a potential underlying cause of unresponsive soft tissue swelling. Timely recognition and accurate diagnosis are paramount to improving patient outcomes in DLBCL, an aggressive lymphoma with a diverse clinical presentation.

## 1. Introduction

Within the head and neck region, lymphoma is the second-most common tumor type after squamous cell carcinoma. Lymphoma can occur in extranodal locations throughout the body and does not necessarily originate in the lymphatic system [1]. Primary cutaneous lymphoma (PCL) is a rare group of non-Hodgkin lymphomas that primarily arise in the skin and typically do not involve lymph nodes or other organs at the time of diagnosis [2]. Among its various subtypes, cutaneous B-cell lymphoma is the most commonly encountered, representing about 20–25% of all primary cutaneous lymphomas [2,3,4]. The 2018 update from the World Health Organization–European Organization for Research and Treatment of Cancer identifies three main subtypes of primary cutaneous B-cell lymphoma (pCBCL): primary cutaneous marginal zone lymphoma (PCMZL), primary cutaneous follicle center lymphoma (PCFCL), and primary cutaneous diffuse large B-cell lymphoma, leg type (PCDLBCL, LT). Among them, primary cutaneous diffuse B-cell lymphoma is a rare form of primary cutaneous B-cell lymphoma, accounting for 10% to 20% of cases and making up only 2% of all primary cutaneous lymphomas [5]. This type of lymphoma is characterized by the rapid growth of reddish or purplish tumor masses. Although diffuse large B-cell lymphoma primarily affects the lower limbs, in 10% to 15% of cases, it may potentially affect areas beyond this region [3]. The rapid growth of this lymphoma is important in differentiating it from other primary cutaneous lymphomas [6]. Nonetheless, it presents with a wide spectrum of clinical features, making accurate diagnosis challenging due to potential overlap with other diseases. Misdiagnosis and delayed recognition of diffuse large B-cell lymphoma can lead to inappropriate treatment and potentially adverse patient outcomes. This study presents a case of diffuse large B-cell lymphoma initially misdiagnosed as a hematoma, highlighting the importance of including malignancies in the differential diagnosis of unresponsive soft tissue swelling.

## 2. Case Report

**Figure 1:**
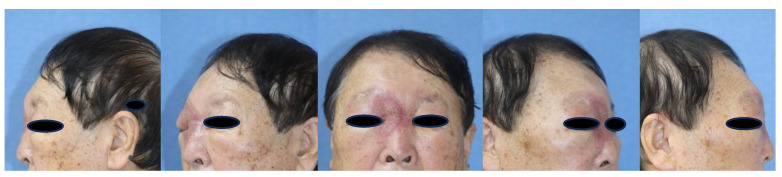
Initial patient picture: right periorbital swelling and ecchymosis.

A 76-year-old male presented to the emergency department with right periorbital swelling and ecchymosis (Figure 1).

**Figure 2:**
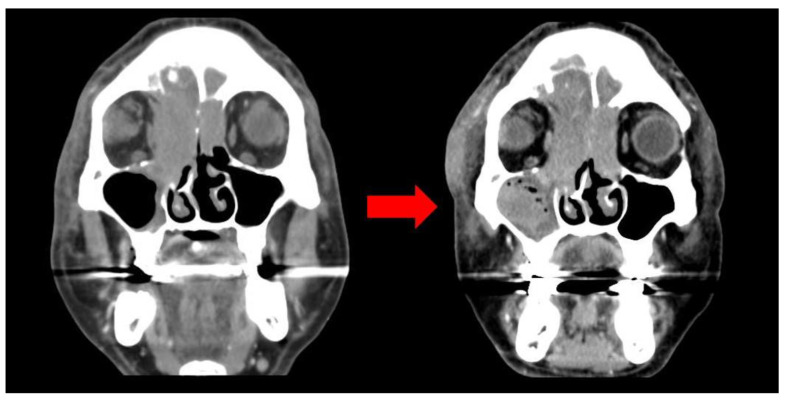
CT image (initial: (**left**); 1 month later: (**right**)): large irregular homogeneous mass in the medial aspect of the right orbit.

These symptoms began 25 days earlier after sustaining an injury to the right orbital region, resulting in a subcutaneous hematoma. The patient had a medical history of hypertension, diabetes mellitus, atrial fibrillation, and cerebral infarction. The patient was taking warfarin for atrial fibrillation and cerebral infarction and denied a family history of malignancy and symptoms including weight loss, fever, and night sweats. The patient underwent trauma at the nasal and periorbital regions by slipping down to the ground, which subsequently resulted in swelling and hematoma. The patient visited a local clinic for evaluation and was prescribed antibiotics and eye drops for a month. But the hematoma persisted despite attempts at aspiration and continued to enlarge. Eventually, it extended to encompass the entire area around the right eye, causing substantial swelling that obstructed the field of vision. A facial bone CT scan exhibited mucoperiosteal thickening with an air-fluid layer in the right maxillary sinus, and bone damage was identified by a large, non-uniform, and homogeneous mass located in the medial aspect of the right orbit and ethmoid region, resulting in the lateral displacement of the eyeball (Figure 2).

A careful ophthalmic exam was performed to determine the necessity of emergent intervention. The initial intraocular pressure of the right eye was 25 mmHg, which was relatively high compared with 16 mmHg of the contralateral eye. But both sides showed negative for rapid afferent pupillary defect (RAPD) and no abnormal sign at pupil size and light reflex. Since extraocular movement of both eyes was relatively well preserved and intraocular pressure of the right eye was at the borderline value, emergent cantholysis for decompression was not performed. Based on the patient’s trauma history and medication history, the author made an impression of the extraconal hematoma involving the medial orbit of the right eye. In response to the request for improved visibility, an incision and drainage (I&D) procedure was performed following anticoagulant bridging therapy. A deep stab incision was made at the medial aspect of the periorbita to drain the hematoma. This resulted in the evacuation of a significant amount of old blood. However, despite continuous drainage, the patient’s symptoms continued to worsen. The swelling involving the right periorbital region became more severe, so the patient could barely open his right eye. A comparison with the previous CT scan revealed a considerable increase in the size of the lesion, which raised suspicion of a malignant tumor. A diagnostic nasal endoscopy was carried out to exclude other illnesses based on the patient’s medical history and radiological findings. Nonetheless, no substantial findings other than mucosal swelling were found. As a result, the otorhinolaryngology department performed a biopsy via endoscopy. No mass-like lesion was observed during the procedure, and histopathological examination revealed chronic inflammation. As such, a surgical excisional biopsy was performed via middle meatal antrostomy (MMA) and inferior neartal antrostomy (INE).

**Figure 3:**
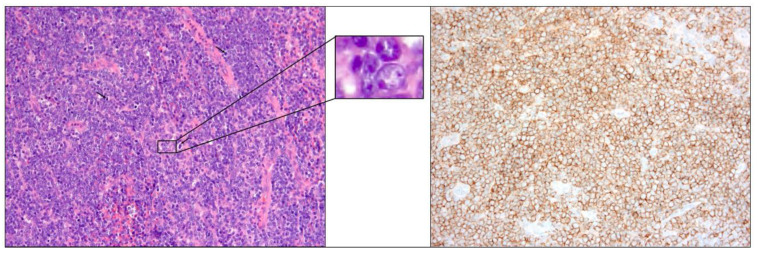
Biopsy slide: diffuse infiltration of homogenous tumor cell (**left**) and CD20 positive (**right**).

The biopsy of the right medial maxillary and ethmoid mass showed lymphocyte dissemination around the tissue without evidence of extracutaneous lymphoma. Tumor cells stained for CD20 and bcl-2 protein expression and did not express CD10 (Figure 3).

A peripheral blood smear was not performed; instead, in collaboration with the hematology department, a bone marrow examination was performed, which confirmed the absence of lymphomatous involvement. The right medial maxillary and ethmoidal mass biopsy confirmed a diffuse large B-cell lymphoma diagnosis. The patient was referred to the hematology department for further management, which encompassed chemotherapy with rituximab, cyclophosphamide, vincristine, doxorubicin, and prednisolone (R-CHOP). Further tests for staging were conducted, and the PET CT/Neck CT results indicated no extracutaneous lymphoma. No evidence of lymphomatous involvement was detected upon bone marrow examination. The patient experienced symptom relief following the initiation of appropriate treatment. After six cycles of chemotherapy, a follow-up PET/CT scan confirmed a slight reduction in the size of previous soft tissue lesions in the upper central face, right lateral face, anterior medial wall of the right orbit, and both ethmoid sinuses.

## 3. Discussion

The orbit is an enclosed space, except for its anterior aspect, which contains various structures, including the eyeball, muscles, ligaments, vasculature, and nerves. Despite the anterior opening, the upper and lower eyelids are strongly supported by ligamentous structures, resulting in a narrow palpebral fissure. Intraorbital hematoma can lead to compression of the eyeball and surrounding structures, causing clinically significant symptoms including disability of ocular movement and vision loss, which often called orbital compartment syndrome. While this condition can arise from ophthalmic surgeries, it can also occur due to trauma. The hematoma located at retrobulbar space, which is also called retrobulbar hematoma (RBH), accounts for about half of traumatic blindness [7]. In such cases, immediate clinical evaluation is necessary to assess the functionality of the eye and its surrounding structures, ensuring that there is no impairment. Prolonged elevated pressure can result in permanent damage to eye movements and vision. Therefore, clinicians must be vigilant in making a differential diagnosis when extraconal hematoma is clinically suspected.

If clinical suspicion of orbital compartment syndrome arises, the clinician must make a decision regarding decompression. There are no clear guidelines for the criteria to perform decompression, and the decision is typically based on a comprehensive assessment of clinical findings, including intraocular pressure, vision, ocular movement, and other physical examination findings [8]. Decompression involves relieving pressure within the orbit by dividing the inferior canthal tendon, which supports the lower eyelid, allowing the eyeball to move forward.

In this case, the patient was taking Warfarin, a potent anticoagulant widely used in medical practice. It is known to increase the risk of hematoma formation, and reports exist of nontraumatic spontaneous retrobulbar hemorrhages associated with its use [9]. The patient initially presented with swelling following facial trauma, which progressively worsened and did not resolve, leading to clinical suspicion of hematoma formation. During incision and drainage, a significant amount of old blood was evacuated, contributing to the delayed diagnosis of lymphoma. In other words, it appears that the formation of a superimposed hematoma may have masked the presence of lymphoma.

Lymphomas are a spectrum of syndromes resulting from the clonal expansion of hematopoietic and lymphoid cells at various developmental stages [10,11]. In roughly 90% of cases, these lymphomas arise from the neoplastic proliferation of B-cells [10,11]. In the head and neck region, malignant lymphomas tend to mainly manifest as lymphadenopathy. Among non-Hodgkin lymphomas, roughly 24% to 48% have extranodal origins, with 8% of this group arising in the ocular adnexa [1,12].

Diagnosis of diffuse large B-cell lymphoma requires histopathological evaluation, which identifies unique features including dense and diffuse infiltration of non-epithelial cells in the skin and subcutaneous tissues [1,12]. The lymphoma cells typically demonstrate expression of CD20, Bcl-2, CD79a, PAX5, IRF4/MUM1, and FOXP1. CD20, Bcl-2, CD79a, PAX5, IRF4/MUM1, and FOXP1 are typically expressed by the lymphoma cells. A poorer prognosis is linked to increased expression of post-germinal center markers such as IRF4/MUM1 and FOXP1 [13]. It is recommended to conduct Epstein–Barr encoding region (EBER) testing in order to exclude Epstein–Barr virus-associated DLBCL. Bcl-6 expression may vary, while CD 10 is negative. Additionally, for diagnostic and staging determination, as well as the exclusion of systemic diseases and treatment, imaging procedures such as PET/CT, laboratory tests, including complete blood count and differential count, and a bone marrow biopsy are recommended.

Treatment options include radiation therapy and chemotherapy. For patients with a single lesion, local radiation therapy is the recommended choice [5]. Patients with multifocal skin lesions or relapses should undergo chemotherapy using the CHOP regimen. This treatment consists of cyclophosphamide, doxorubicin, vincristine, and prednisone, and is considered the standard chemotherapy for this disease. The response rate for this treatment typically ranges from 40% to 50%. As with all lymphomas, it is advisable to screen for HCV antibodies, HBV surface antigens, and HBV core antibodies before initiating each chemotherapy protocol [13]. This is performed to detect any potential viral reactivation and to take appropriate prophylactic measures [13]. Although patients who undergo a combination of chemotherapy and radiation may achieve better clinical outcomes than those treated with only chemotherapy, it should be noted that many DLBCL patients are elderly and frail, making them unsuitable candidates for chemotherapy [14,15]. Furthermore, despite chemotherapy and radiation therapy, recurrence and systemic spread are prevalent [16,17]. This situation adds complexity to the treatment of primary cutaneous large B-cell lymphoma. After the initial treatment, the follow-up consists of a comprehensive clinical skin and lymph node examination, laboratory tests, and PET CT evaluations, coupled with annual restaging [18]. After concluding the treatment, PET CT scans are mandatory every 6–12 months.

Although primary cutaneous DLBCL is a rare occurrence, it carries an unfavorable prognosis. The overall 5-year survival rate for DLBCL ranges from 20% to 55% [2]. The number of skin lesions at the moment of diagnosis is essential in assessing these patients. A comprehensive evaluation of lymph nodes and extracutaneous conditions is essential for patients with diffuse large B-cell lymphoma.

The etiological factors that contribute to NHL are diverse and include immunodeficiency, viral infections such as the Epstein–Barr virus and radiation exposure, hybrid genes resulting from translocation, and radioactive contamination. While several reports have documented cases where lymphoma was diagnosed 1 to 2 months after trauma, the etiological role of trauma in the onset of lymphoma is not well understood [18,19,20]. Barnett et al. [21] suggested that prolonged or recurrent inflammation resulting from trauma may induce cellular atypia, potentially leading to the development of neoplasia. Additionally, previous facial trauma may create a locus minoris resistentiae where circulating lymphoma cells can accumulate and become trapped, potentially leading to the formation of lymphoma nodules [20]. While the connection between the patient’s trauma and the tumor’s onset remains unclear, our case can serve as an example of facial lymphoma arising as a result of trauma.

Initially, the absence of typical lymphoma-related symptoms, such as night sweats, weight loss, and fatigue, posed a diagnostic challenge. Moreover, the patient was undergoing long-term warfarin therapy for atrial fibrillation. Initially, the examination of nasal endoscopy tissue found no particular lesions except mucosal swelling. However, suspicion of malignancy was warranted due to the detection of bone erosion and mass effects on CT scans, as well as the observation of a fast-growing mass, despite the lesion persisting beyond the usual one-month healing period. Recognizing that early diagnosis and treatment can significantly improve patient survival rates, it is essential to consider malignancy as a possible cause when dealing with unresponsive soft tissue swelling.

In addition, it’s important to note that while diffuse large B-cell lymphoma (DLBCL) is the focus of this discussion, T-cell lymphomas also play a significant role in the spectrum of lymphomas. For example, anaplastic large cell lymphoma (ALCL) is a T-cell lymphoma that should be considered in the differential diagnosis.

ALCL is characterized by large, anaplastic lymphoid cells and can occur in both cutaneous and systemic forms. The cutaneous form often presents as skin lesions. Primary cutaneous ALCL makes up 8% of CTCLs [22], is usually indolent, with frequent relapse, and has an excellent prognosis [23]. It is seen mostly in adults, rarely in children, with males diagnosed more often than females [24].

T-cell lymphomas (TCLs) have a lower frequency than B-cell lymphomas. TCLs are a heterogeneous group of diseases further subdivided into peripheral T-cell lymphomas (PTCLs) and cutaneous T-cell lymphomas (CTCLs) [24]. PTCL generally refers to a group of TCLs that arise from mature T cells after they leave the thymus. Most subtypes of TCLs are considered PTCL, with CTCL being relatively rare, accounting for only 2–3% of all non-Hodgkin’s lymphoma (NHL) cases. CTCLs usually manifest on the skin due to the expression of cutaneous lymphocyte antigen [25].

Anaplastic large cell lymphoma (ALCL) and peripheral T-cell lymphoma, not otherwise specified (PTCL-NOS) are important subtypes of PTCL to be distinguished from this case. Recognizing the difference between B-cell lymphoma and T-cell lymphoma is critical to an accurate diagnosis and an appropriate treatment plan. Thus, a comprehensive differential diagnosis is imperative when assessing a patient with lymphoma, including the possibility of T-cell lymphoma in addition to B-cell lymphoma, to prevent delayed treatment.

## 4. Conclusions

In summary, diffuse large B-cell lymphoma (DLBCL) is a rare disease associated with a poor prognosis. When considering DLBCL, it is important to recognize that cutaneous lymphoma can manifest with diverse and atypical symptoms, such as hematomas. This case highlights the importance of considering cutaneous lymphoma as a potential diagnosis when certain clinical features are present.

In particular, a history of trauma, as seen in this case, can be a confounding factor. When a hematoma persists for more than one month and does not respond to appropriate treatment, it should raise suspicion for underlying malignancies, including cutaneous lymphoma.

This case highlights the importance of early and accurate diagnosis, especially in rare and challenging conditions such as primary cutaneous DLBCL. Timely recognition and a multidisciplinary approach to treatment are critical to improving outcomes for patients with this aggressive form of lymphoma.

## Data Availability

Data is contained within the article. The data presented in this study are available in this article here.
